# Novel, *Meso*-Substituted Cationic Porphyrin Molecule for Photo-Mediated Larval Control of the Dengue Vector *Aedes aegypti*

**DOI:** 10.1371/journal.pntd.0001434

**Published:** 2011-12-20

**Authors:** Leonardo Lucantoni, Michela Magaraggia, Giulio Lupidi, Robert Kossivi Ouedraogo, Olimpia Coppellotti, Fulvio Esposito, Clara Fabris, Giulio Jori, Annette Habluetzel

**Affiliations:** 1 School of Pharmacy, University of Camerino, Camerino, Italy; 2 Department of Biology, University of Padova, Padova, Italy; 3 School of Bioscience and Biotechnology, University of Camerino, Camerino, Italy; 4 Institut de Recherche en Sciences de la Santé (IRSS), Bobo-Dioulasso, Burkina Faso; Mahidol University, Thailand

## Abstract

**Background:**

Control of the mosquito vector population is the most effective strategy currently available for the prevention of dengue fever and the containment of outbreaks. Photo-activated oxidants may represent promising tools for developing effective, safe and ecofriendly novel larvicides. The purpose of this study was to evaluate the potential of the synthetic *meso*-substituted porphyrin meso-tri(N-methylpyridyl), meso-mono(N-tetradecylpyridyl)porphine (C14) as a photoactivatable larvicide against the dengue vector *Aedes (Stegomyia) aegypti*.

**Methodology:**

The photophysical and photochemical properties of the C14 molecule were assessed spectrophotometrically. Photomediated larvicidal efficacy, route of intake and site of action were determined on *Ae. aegypti* larvae by laboratory bioassays and fluorescence microscopy. Using powdered food pellet for laboratory rodents (a common larval food used in the laboratory) as a carrier for C14, loading-release dynamics, larvicidal efficacy and residual activity of the C14-carrier complex were investigated.

**Main Findings:**

The C14 molecule was found to exert a potent photosensitizing activity on *Ae. aegypti* larvae. At irradiation intervals of 12 h and 1 h, at a light intensity of 4.0 mW/cm^2^, which is 50–100 times lower than that of natural sunlight, LC_50_ values of 0.1 µM (0.15 mg/l) and 0.5 µM (0.77 mg/l) were obtained, respectively. The molecule was active after ingestion by the larvae and caused irreversible, lethal damage to the midgut and caecal epithelia. The amphiphilic nature of C14 allowed a formulate to be produced that not only was as active against the larvae as C14 in solution, but also possessed a residual activity of at least two weeks, in laboratory conditions.

**Conclusions:**

The *meso-*substituted synthetic porphyrin C14, thanks to its photo-sensitizing properties represents an attractive candidate for the development of novel photolarvicides for dengue vector control.

## Author Summary

Dengue is a life-threatening viral disease of growing importance, transmitted by *Aedes* mosquito vectors. The control of mosquito larvae is crucial to contain or prevent disease outbreaks, and the discovery of new larvicides able to increase the efficacy and the flexibility of the vector control approach is highly desirable. Porphyrins are a class of molecules which generate reactive oxygen species if excited by visible light, thus inducing oxidative cell damage and cell death. In this study we aimed at assessing the potential of this photo-mediated cytotoxic mechanism to kill *Aedes (Stegomyia) aegypti* mosquito larvae. The selected porphyrin molecule, meso-tri(N-methylpyridyl),meso-mono(N-tetradecylpyridyl)porphine (C14 for simplicity), killed the larvae at doses lower than 1 µM, and at light intensities 50–100 times lower than those typical of natural sunlight, by damaging their intestinal tissues. The physicochemical properties of C14 make it easily adsorbed into organic material, and we exploited this feature to prepare an ‘insecticidal food’ which efficiently killed the larvae and remained active for at least 14 days after its dispersion in water. This study demonstrated that photo-sensitizing agents are promising tools for the development of new larvicides against mosquito vectors of dengue and other human and animal diseases.

## Introduction

Mosquitoes belonging to the *Aedes* genus are vectors of several human arboviruses, the most important of which is the dengue virus. Global dengue incidence has grown dramatically in the last decades, favoured by increased human mobility and urbanization [1]; currently, 2.5 billion people worldwide live at risk of dengue, mainly in tropical countries [2]. *Aedes (Stegomyia) aegypti* represents the main dengue vector species, because of its marked anthropophily and for its ability to proliferate in close proximity with human communities by using artificial water storages such as tanks, drums, buckets, flower vases, etc. as breeding sites [3]–[4].

Since a vaccine against dengue is still lacking, control of the mosquito vector density is considered the most effective strategy for the prevention of the disease [5]. Integrated vector management (IVM), coupled with virus surveillance, can contain dengue outbreaks and limit dengue cases and fatalities [6]. Specifically, interventions targeted to the larval stages of the vector, consisting of larvicide treatments, water source reduction and/or use of natural predators, are crucial components of successful dengue prevention and control programmes [7]–[8].

During the last decades, the broad use of organophosphates (mainly temephos) for the control of *Ae. aegypti* larvae and adults led to the emergence of insecticide resistance in many dengue endemic countries [9]–[11]. Currently, chemical control of *Aedes* larvae is being largely achieved through the use of the insect growth regulators methoprene and pyriproxyfen, and the biopesticide *Bacillus thuringiensis israelensis* (Bti), all of which can be safely applied in water storages for domestic use [3]. Additionally, the biopesticide spinosad can be effectively used in water collections not intended for drinking purposes [3], and monomolecular films (MMF), such as Agnique®, represent a potentially interesting alternative to chemical insecticides, for their ability to kill mosquito immature stages by non-chemical means [12]. Several factors pose challenges to the use of the available larvicides in the control of dengue vectors, including the diversity of productive breeding sites and the economic and cultural differences among affected communities which result in different patterns of domestic water availability/management [13]–[14] and of acceptance of vector control interventions [7], [15]. Therefore, the development of larvicides based on novel mechanisms of action is desirable to complement the existing tools and increase the scope of dengue vector control. Ideally, these products should not induce resistance, thus allowing for an increased lifespan, they should be based on inexpensive active principles and formulation materials, be easy to handle/apply and possess a good residual activity. Most importantly, they should be safe for humans and non-target organisms sharing the same habitat as the larvae, e.g. predators used as biological control agents, and their use should be well accepted by the recipient communities.

Photo-activated processes may profoundly affect biological systems [16]. Frequently, these reactions involve a highly reactive singlet oxygen (^1^O_2_) intermediate, generated via energy transfer from a photoexcited sensitizer, as the main biotoxic agent [17].

Several strategies have been developed to drive the photosensitizer to specific locations in cells and tissues. Currently available techniques are based on the molecular engineering of the photosensitizer, in order to enhance its affinity for selected constituents of the target. The short lifetime of the reactive oxygen intermediate (in the nano- to micro-second range) limits its phototoxic action to the immediate proximity of the photoexcited molecule; on the other hand, the high reactivity of singlet oxygen typically results in a large diversity of cell constituents being oxidized, thus granting a multi-targeted action, which *per se* reduces the likelihood of selecting for target site resistance.

Several synthetic and naturally derived photosensitizers, such as thiophenes, xanthenes, phenothiazines, furocoumarins, acridines, tetraethynylsilanes and porphyrins, have been demonstrated to be effective photoinsecticides against a range of noxious insects, including flies [18]–[19] and mosquitoes [20]–[23]. In particular, porphyrin derivatives, such as chlorophyllin, pheophorbid and hematoporphyrin, exhibit a marked larvicidal activity on *Culex* and *Aedes* in the laboratory as well as in semi-field conditions [24]–[28].

The use of porphyrin photosensitizers as mosquito larvicides is comparably safer than conventional chemicals. Their activity being dependent on photoactivation by visible light, they are harmless in case of ingestion by humans and, in general, by non-translucent organisms. Furthermore, porphyrins undergo gradual photobleaching in the sunlight and their photodegradation products do not induce appreciable toxic effects in a variety of biological systems [29].

Among porphyrin derivatives, *meso*-substituted porphyrins are particularly advantageous, allowing the physico-chemical and photobiological properties of the photosensitizer to be tailored to the biochemical and physiological features of the target, and its partitioning among subcellular or subtissular compartments to be finely tuned [17].

The objective of this study was to evaluate the potential of a newly developed synthetic *meso*-substituted cationic porphyrin (C14) as a photosensitizing agent for the control of mosquito larvae. The photophysical and photochemical properties of the molecule, and its insecticidal efficacy on *Ae. aegypti* larvae were determined. In addition, a model C14 formulate obtained using powdered food pellet for laboratory rodents (a commonly used mosquito larval food in the laboratory) as a porphyrin carrier, was analysed in terms of C14 loading-release dynamics, photoinsecticidal efficacy and residual activity.

## Materials and Methods

### Ethics statement

Laboratory rodents (BALB/c mice) were used to provide blood meals to mosquitoes for the maintenance of the colony. Animal rearing and handling (licence no. 125/94A, issued by the Italian Ministry of Health) were fully compliant with the Italian Directive 116 of 10/27/92 on the “use and protection of laboratory animals”, and in adherence with the European regulation (86/609) of 11/24/86.

### C14 porphyrin molecule

**Figure 1:**
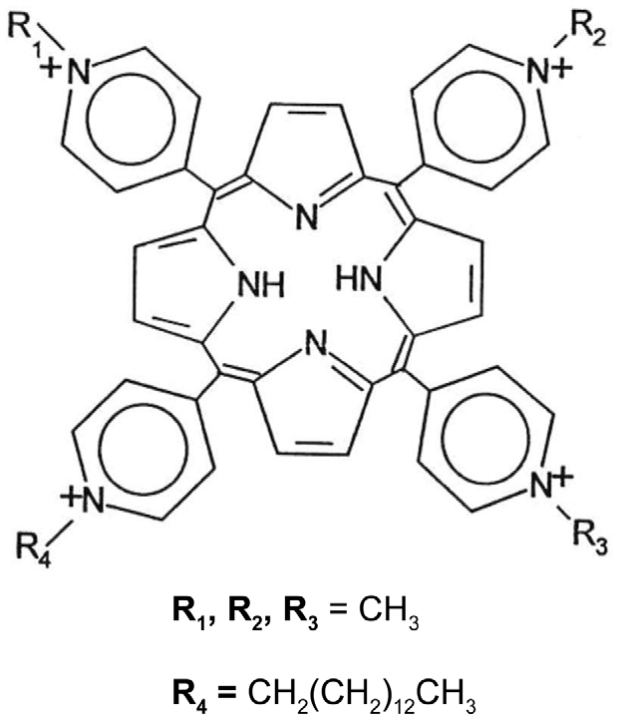
Chemical structure of C14 porphyrin: meso-tri(N-methylpyridyl),meso-mono(N-tetradecylpyridyl)porphine.

As photosensitizer, a synthetic cationic porphyrin, meso-tri(N-methylpyridyl),meso-mono(N-tetradecylpyridyl)porphine tetrasulphonate (MW = 1545,96) was used (Figure 1). The porphyrin, hereafter referred to as C14 for simplicity, was kindly supplied by Dr. J. Bommer from Frontier Scientific Inc., Logan, UT, USA. The C14 molecule was dissolved in water to obtain stock solutions at concentrations of 50–500 µM, according to the experimental needs.

### Lighting system and conditions of irradiation

All experiments involving irradiation of mosquito larvae were performed by employing full spectrum visible light (400–800 nm) at a fluence rate of 1.0 to 4.0 mW/cm^2^ using low-pressure mercury discharge fluorescent tubes TL-D Standard Colours (TL-D 58W/33-640 1SL, PHILIPS, EC). The intensity of the incident radiation was measured by an ILT1400A radiometer/photometer, equipped with a SED623/HNK15 multi-junction thermopile detector (International Light Technologies Inc., MA, USA).

### Mosquito colony maintenance

The *Ae. aegypti* mosquito colony was established from eggs obtained from the Swiss Tropical Institute (Basel, Switzerland). The colony was maintained at 28±2°C, >90% RH and a photoperiod of 12 h, in a climatic chamber within a double door access insectary, devoid of windows. The utmost care was taken throughout the study to avoid accidental releases of mosquitoes. Larvae were fed with ground food pellet for laboratory rodents (Mucedola Srl, Italy). A 5% sucrose solution in soaked cotton pads was offered to adults *ad libitum*.

### Photophysical and photochemical studies

#### Photostability in the aqueous medium

The photostability of the C14 porphyrin was determined in phosphate-buffered saline (PBS) upon illumination of a 2.5 µM porphyrin solution (initial absorbance around 0.5 at 424 nm) with white light (400–800 nm), which was isolated from the emission of a quartz-halogen lamp equipped with broad band filters to eliminate UV and infrared radiation. The light source was supplied by Teclas (Lugano, Switzerland), and operated at a fluence rate of 20 mW/cm^2^. During irradiation the porphyrin solution was kept in agitation on a magnetic stirrer at room temperature. The concentration of the porphyrin in the aqueous solution was monitored spectrophotometrically at different irradiation times up to 60 min, using a VARIAN spectrophotometer (UV-VIS, Cary 50 Series) and the photostability was expressed as the percent residual absorbance referred to the absorbance measured before irradiation.

#### Determination of singlet oxygen quantum yield

The potential of the C14 porphyrin as a photosensitising agent was assessed on the basis of the quantum yield (φ_Δ_) of singlet oxygen (^1^O_2_) generation by the photoexcited triplet state of the porphyrin, namely the number of ^1^O_2_ molecules generated per number of absorbed light photons. In the present study φ_Δ_ was measured by following the decrease in the fluorescence emission of 9,10-dimethyl-anthracene (DMA) upon its photosensitised conversion into the corresponding non-fluorescent 9,10-endoperoxide. The reaction of singlet oxygen with DMA occurs with 100% chemical quenching (no competing physical quenching), hence the amount of DMA modified in the reaction also provides information on the quantitative yield of singlet oxygen generation [30]. In a typical experiment, a DMA solution (1.5 ml, initial absorbance around 1 at 380 nm) and porphyrin solution (1.5 ml, initial absorbance around 0.4 at 420 nm) in N,N-dimethyl-formamide (DMF) were placed in a quartz cuvette with a 1 cm optical path and irradiated with 400–800 nm light wavelengths (Teclas lamp, 100 mW/cm^2^) at 20±2°C under gentle magnetic stirring for different periods of time up to 20 min (0, 1, 3, 5, 10, 15, 20 s and 1, 3, 5, 7.5, 10, 12.5, 15, 17.5, 20 min). The DMA fluorescence emission was recorded in the 380–550 nm wavelength range with excitation at 360 nm. The first-order rate constant of the photoprocess was obtained by plotting ln *F*_0_/*F* as a function of the irradiation time *t*, where *F_0_* and *F* represent the fluorescence intensity at time 0 and time t, respectively. The slope of the linear plot thus obtained allowed the rate constant of the photoprocess (k) to be calculated. The constant was then converted into ^1^O_2_ quantum yield using the following proportion:
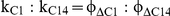
where k_C1_ is the rate constant for DMA photooxidation sensitized by C1 porphyrin, an analogue of C14 with a methyl group in place of the tetradecyl chain, used here as a reference compound. The φ_ΔC1_ was shown to be 0.51 [31].

### Formulation studies

A standard food pellet for laboratory rodents, namely 4RF18 GLP (Mucedola Srl, Italy), commonly used in our laboratory as mosquito larval food, was crushed using an electric blender and then sieved (mesh size 500 µm) to obtain powdered food pellet (PFP) with final particle size of 5–500 µm diameter. C14-PFP complexes were obtained by incubating PFP in C14 solutions. The loading of C14 on PFP and the dynamics of its release from the C14-PFP complexes in water were analysed by spectrophotometric quantification. Specifically, to evaluate the C14 binding rate on PFP, 70 mg of PFP were incubated in 500 ml of a 5 µM C14 solution, at 28°C for 5 days, in the dark. The same C14 solution without PFP served as control. The amount of unbound porphyrin was then estimated by measuring the absorbance at 423 nm of the supernatant of solution aliquots collected at various times during incubation and centrifuged at 10,000 rpm for 10 min. To test the stability of C14-PFP complexes in aqueous media, 6 mg of the formulate, containing 72 µg of C14, were incubated at 30°C in 100 ml of buffered solutions at 4 different pH values in the presence of light. The following buffers were used: 50 mM potassium phosphate buffer (pH 7.0 and 7.6), 50 mM Tris-HCl buffer (pH 8.4) and 50 mM glycine-NaOH (pH 9.5). The amount of porphyrin released in the media from the formulate complexes was measured as described above.

To assess the effect of different C14 loading concentrations on the photolarvicidal activity of the C14-PFP complexes, two formulates (C14PF-5 and C14PF-50) were prepared by incubating 25 mg of PFP in 500 ml of 5 µM and 50 µM C14 aqueous solutions overnight at room temperature under gentle shaking. After incubation, the solutions were filtered using Whatman qualitative filter papers (Whatman International Ltd., UK) and the solid residues, consisting of the C14-PFP complexes, were washed with 10 ml distilled water, oven dried at 37°C for 4 h and stored at room temperature until use. To quantify the amount of bound C14, samples of the two C14-PFP formulates were dissolved in 3 ml of 2% SDS for 2 h under gentle magnetic stirring. The extracted porphyrin was then quantified by spectrophotometric analysis as described above.

### Larvicidal activity experiments

All the experiments were carried out in a climatic chamber at 28±2°C with a regulated photoperiod of 12 h. Transparent plastic trays, containing 500 ml C14 solutions or spring water were employed. Typically, 5±1 days old, 3^rd^–early 4^th^ instar *Ae. aegypti* larvae were used. All the experiments were replicated three times.

#### C14 porphyrin toxicity in the dark

To assess whether C14 is endowed with an intrinsic toxicity in the absence of irradiation, four groups of about 50 larvae (range 48–61) each were introduced into three trays containing 5 µM C14 solutions and one tray containing spring water as a control, all of which were provided with PFP. Trays were incubated in the dark for 3, 8 or 24 h. At the end of the incubation period, dead and living larvae were counted in each tray. Larvae were then washed with tap water, and transferred to clean trays containing spring water and PFP. Trays were kept in the dark until adults emerged. Adults were counted and exposed to light (intensity 1.0–4.0 mW/cm^2^) for 12 h, and their mortality was evaluated at the end of the irradiation period.

#### C14 porphyrin phototoxicity

Groups of 50 larvae each were added to trays containing 5 µM porphyrin C14 solution or spring water as a control. Trays were provided with PFP and incubated in the dark for 12 hours. After the dark incubation period, two C14-containing trays were irradiated for 1 hour and 6 hours, respectively, at a light intensity of 1.0–4.0 mW/cm^2^, while the control tray was irradiated for 6 hours. After irradiation, all the trays were returned in the dark, and larval mortality was assessed every 24 hours for the following 6 days. Larvae not moving or not showing a normally vigorous escaping response at probing were defined as dead or dying, respectively, and counted together. Pupae formed during the experiment were transferred to smaller trays containing spring water, within screened cages at normal colony photoperiod conditions, and monitored for mortality and adult emergence.

#### Larvicidal efficacy of C14 porphyrin

Trays containing C14 porphyrin solutions at 7 increasing concentrations (range 0.03–4.3 µM), or spring water as a control, were provided with 6 mg of PFP each and then incubated in the dark for 48 hours. Batches of 100 larvae, deprived of food for the previous 24 hours, were introduced into the trays at 8.00 pm (beginning of the 12 h-long dark period in the climatic chamber). Larval mortality was evaluated on the next day at 9 am (after 1 hour irradiation), 2 pm (6 hours irradiation) and 8 pm (12 hours irradiation). An additional mortality evaluation was performed on the following day at 8.00 am, after a further overnight incubation in the dark. Larvae not moving or not showing a normally vigorous escaping response at probing were defined as dead or dying, respectively, and counted together. Pupae occasionally formed during the experiment, which never exceeded 10% of the total number of larvae, were discarded and excluded from the evaluation.

#### Larvicidal efficacy of C14 porphyrin-loaded PFP

Trays containing 5 µM C14 porphyrin solution and 70 mg PFP were incubated at 28±2°C for 5 days in the dark. The solution was then filtered using Whatman qualitative filter papers (Whatman International Ltd., UK). The eluted C14 solution was conserved, and the incubated PFP retained on the filter paper was washed with 10 ml distilled water before further use. Experimental groups were designed as follows: 1) filtered, C14-incubated PFP in spring water (group A); 2) C14 solution eluate, added with 70 mg fresh PFP (group B); 3) 5 µM C14 solution incubated without PFP for 5 days in the dark, added with 70 mg fresh PFP (group C); 4) freshly prepared 5 µM C14 solution, added with 70 mg fresh PFP (group D); 5) spring water added with 70 mg fresh PFP (control group). Fifty larvae, fasting for 24 hours, were added to each tray and incubated in the dark for 12 hours. Treated or untreated PFP was added at the time of introduction of the larvae, in all the experimental groups. The trays were then exposed to a light intensity of 1.0–4.0 mW/cm^2^ and dead, dying and living larvae were counted 1 to 3 hours after the beginning of the irradiation.

#### Fluorescence microscopy

Additional samples of larvae were exposed to the same conditions as described in the above experiment and examined at the fluorescence microscope to determine C14 localization in the body after uptake and to observe organ morphology. Samples of treated and untreated PFP were also examined to qualitatively assess C14 adsorption. A Zeiss Axio Observer Z1 (Carl Zeiss AG, Oberkochen, Germany) at 50×–400× magnification in fluorescence light, and a FITC09 filter (excitation bandpass 450–490 nm; emission longpass 515 nm) were used.

#### Efficacy and residual activity of photolarvicidal formulations

Five series of trays were prepared, namely: 0.3 µM and 5 µM C14 porphyrin solutions containing 6 mg PFP; spring water containing 6 mg C14PF-5 or C14PF-50 formulate and spring water containing 6 mg PFP as a control. Each series was arranged into three groups, which were incubated at a 12 hour photoperiod for 48 hours, one or two weeks, respectively. At the end of the incubation periods, batches of 100 larvae, fasting for 24 hours, were introduced into the trays at 8.00 pm (beginning of the 12 h-long dark period in the climatic chamber). Larval mortality was evaluated after 12 hours of irradiation (8.00 pm next day).

#### Statistical analysis

Mortality data were analyzed by ANOVA. Comparisons of the mean mortality values of multiple treatment groups were obtained by attributing different letter codes to significantly different means on the basis of the Fisher's Least Significant Difference (LSD) post-hoc test [32]. ANOVA and LSD tests were performed using SPSS v. 11.0 (SPSS Inc.). LC_50_ values and relevant statistics were obtained by means of nonlinear regression (fitting of 4-parameters logistic equation) using OriginPro v. 7.5 (OriginLab Corp.).

## Results

### Photophysical and photochemical studies

**Figure 2:**
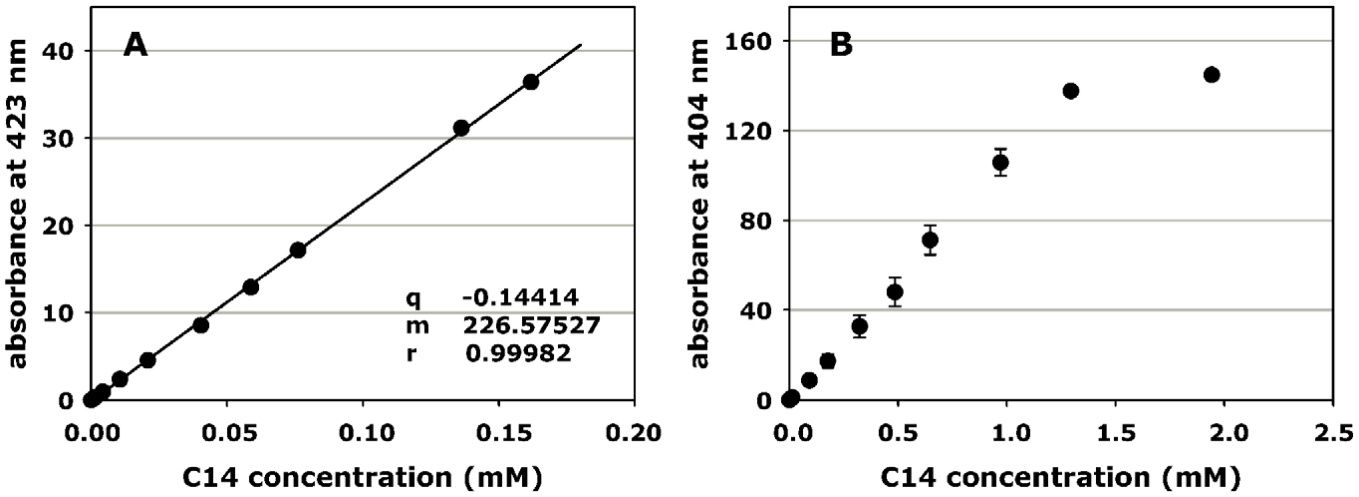
Effect of concentration on the absorbance of C14 porphyrin solutions. Solutions were prepared in PBS. **A:** absorbance of solutions at the maximum of the Soret band (424 nm); **B:** absorbance at a wavelength characterized by a lower molar extinction coefficient (404 nm).

When dissolved in neutral aqueous solution, the C14 porphyrin exhibited the typical absorption spectrum of *meso*-substituted porphyrin derivatives, and in particular the maximum absorbance of the intense Soret band was located at 424 nm. To test the possible occurrence of aggregation processes for this porphyrin, the intensity of the Soret band was titrated as a function of the porphyrin concentration according to the Beer-Lambert law. In a first phase of our investigations, the data were calculated up to a porphyrin concentration of 0.16 mM (Fig. 2 A), since the optical density of more concentrated porphyrin solutions became too large even using cuvettes of 0.1 cm optical path. While the strictly linear plot would indicate that C14 exists in a purely monomeric state up to 0.16 mM in aqueous solution, an attentive observation of the shape of the absorption spectrum (data not shown) suggests that a slight shoulder on the shorter wavelength side of the C14 Soret band appears at the highest concentration investigated by us. This spectral feature is generally attributed to the presence of porphyrin oligomers [31]. To test the possibility that the hydrophobicity imparted by the long alkyl chain of C14 may favour the occurrence of some aggregation as the concentration increases, the titration was extended to larger molarities calculating the absorbance values at 404 nm instead of 424 nm (Fig. 2 B). The plot for C14 clearly deviates from linearity at porphyrin concentrations between 1.0 and 1.5 mM, indicating that this porphyrin aggregates in this concentration range.

#### Photostability in the aqueous medium

The stability of C14 to the effect of full spectrum visible light was studied for a 2.5 µM porphyrin solution in PBS. The exposure of the porphyrin to visible light at a fluence rate of 20 mW/cm^2^ for up to 60 min caused a decrease in the overall absorbance of less than 10%, which involved the whole set of bands in the blue, green and red spectral region. Therefore, this porphyrin appears to be endowed with a marked photostability, taking into account that most porphyrins undergo a 50% or larger photodegradation under similar irradiation conditions [16].

#### Determination of singlet oxygen quantum yield

**Figure 3:**
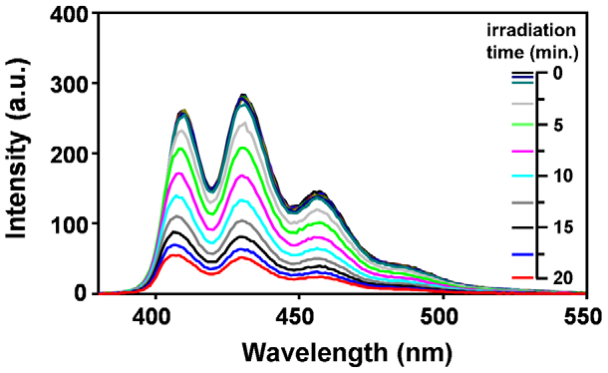
Efficiency of singlet oxygen generation by photoactivated C14 porphyrin. Effect of the irradiation time on the fluorescence properties of a DMA solution (initial absorbance around 1 at 380 nm) and porphyrin solution (initial absorbance around 0.4 at 420 nm) in N,N-dimethyl-formamide (DMF), which was exposed to white light (400–800 nm) at a fluence rate of 100 mW/cm^2^. The spectra taken at 1, 3, 5, 10 and 15 s were overlapping, and the corresponding coloured lines have been omitted from the legend, for clarity.

It is known [16], [33] that porphyrin photosensitisation of biological systems largely proceeds via generation of singlet oxygen (^1^O_2_), a highly reactive oxygen derivative, as the most toxic intermediate. The quantum yield of ^1^O_2_ generation by the photoexcited C14 porphyrin was determined by a chemical quenching method, using 9,10-dimethyl-anthracene (DMA) as a target. A typical time-dependence of the photoinduced decrease in the fluorescence emission of DMA upon increasing irradiation times in the presence of C14, due to the conversion of the polycyclic aromatic derivative to its non-fluorescent 9,10-endoperoxide was obtained (Fig. 3). The emission spectrum of DMA is characterized by the presence of three main bands in the 400–500 nm wavelength interval, all of which showed an identical rate of photoinduced decrease. The quantum yield of ^1^O_2_ photogeneration by C14 was found to be 0.46. Therefore, about 50% of the C14-absorbed photons are conveyed to the direct promotion of the photosensitised oxidative processes that elicit damages to cells and tissues.

### Formulation studies

**Figure 4:**
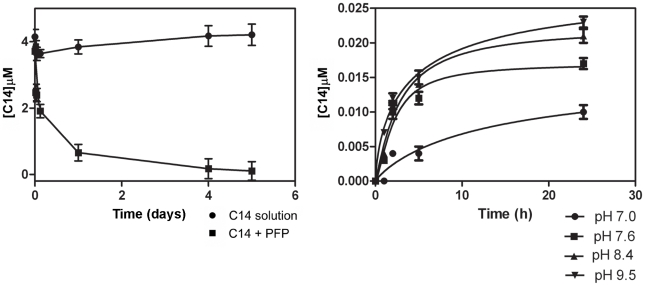
Adsorption and release dynamics of C14 on PFP (particle size 5–500 µm). **A**: Residual concentration of C14 (5 µM) as function of incubation time at 28±2°C in the presence (▪) and in absence (•) of PFP (70 mg/500 ml C14 solution); **B**: stability of the C14-loaded PFP at various pH values.

PFP incubated in a 5 µM C14 solution efficiently adsorbed the compound and sequestered it from the solution. Already 24 h after the beginning of the incubation, 82% of C14 porphyrin was bound to the PFP particles, and its amount increased to reach 92% after 5 days of incubation, while no appreciable decrease in concentration was observed in a C14 solution incubated under the same conditions without PFP (Fig. 4 A). C14 appeared to remain stably associated with PFP when C14-loaded PFP particles were incubated for 24 h in C14-free buffer solutions. Specifically, the porphyrin concentrations in the incubation buffers ranged from 0.01 µM (pH 7.0) to 0.024 µM (pH 9.5), corresponding to a release of just 2.14%–5.15% of the initial C14 amount (Fig. 4).

### Larvicidal activity experiments

#### C14 porphyrin toxicity in the dark

No mortality was detected on *Ae. aegypti* larvae incubated in a 5 µM C14 solution in the dark, irrespectively of the duration of incubation (Table 1). All the exposed larvae pupated normally (data not shown), and the proportion of adults emerged was comparable to that of untreated controls (Table 1). No mortality was observed after the adults were exposed to light. The experiment demonstrates lack of toxicity by C14 in the dark, and lack of delayed effects on emerged adults.

**Table 1:** Survival of *Ae. aegypti* larvae exposed to C14 in the dark.

incubation time (h)^a^	larval % survival^b^	proportion of emerged adults (%)^c^	adult % survival after light exposure^d^
3	100 (0.0)	138/141 (97.9)	100
8	99.3 (1.2)	128/128 (100)	100
24	100 (0.0)	138/141 (97.9)	100
control	99.4 (1.1)	155/157 (98.7)	100

a incubation was carried out with 5 µM C14 in the dark at 28±2°C; control larvae were incubated for 24 h.

b surviving larvae at the end of the incubation period (n = 3 replicates of ∼50 larvae each). Numbers in parentheses indicate standard deviations.

c Emerged adults/total pupae; pooled data from the three replications.

d 12 h-long irradiation (1.0–4.0 mW/cm^2^).

#### C14 porphyrin toxicity in the light

**Figure 5:**
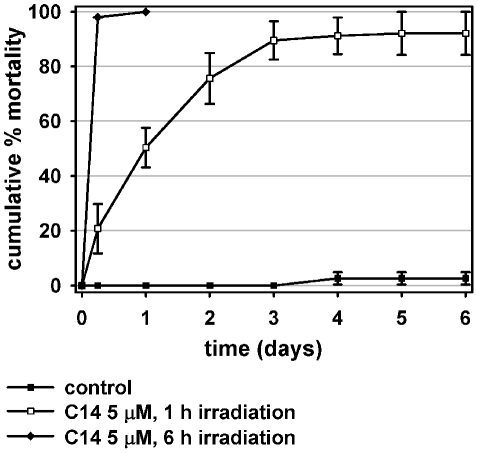
Larvicidal photosensitizing effect of C14 porphyrin. Mortality of *Ae. aegypti* larvae (n = 50) incubated with C14 at 28±2°C in the dark for 12 h, and then exposed to light (fluence rate 1.0–4.0 mW/cm^2^) for 1 or 6 h. After irradiation, larvae were kept in the dark and larval mortality was monitored daily for 6 days. Arithmetic means of % dead larvae. Error bars represent standard deviation (n = 3 replicates of 50 larvae each).

A 6 h exposure to light (fluence rate 1.0–4.0 mW/cm^2^) of larvae previously dark-incubated for 8 h in a 5 µM C14 solution determined an almost complete mortality within the irradiation period (Fig. 5) and mortality reached 100% at the following count, carried out 24 h later. The photosensitizing effect was irreversible, as demonstrated by the mortality of treated larvae irradiated for 1 h and thereafter kept in the dark, which showed a continuous increase during the days following irradiation, and reached 92.1%±7.9% on day 6 (Fig. 5).

#### Larvicidal efficacy of C14 porphyrin

**Figure 6:**
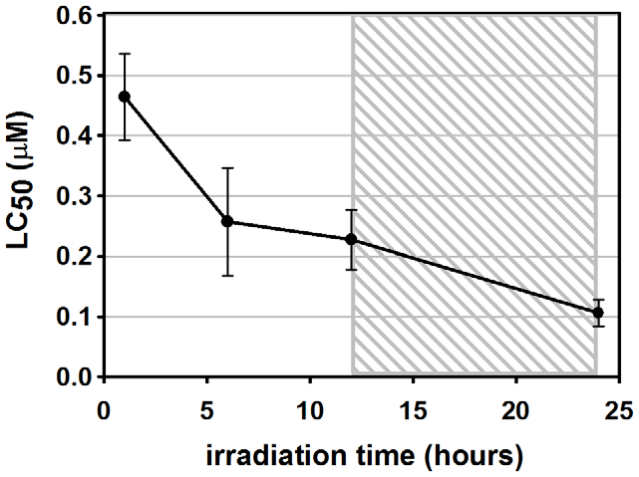
Influence of irradiation time on C14 porphyrin LC_50_ on *Ae. aegypti* larvae. Error bars represent 95% confidence interval (n = 3 replicates of 100 larvae each). The shaded area in the graph indicates the period of incubation without light (night).

The LC_50_ values of C14 on 3^rd^–4^th^ instar *Ae. aegypti* larvae showed an inverse relationship with the irradiation time (Fig. 6). After 1 h irradiation at a fluence rate of 1.0–4.0 mW/cm^2^, the C14 LC_50_ was 0.46 µM (Table 2), and its value halved after 12 h irradiation. An additional overnight incubation in the dark of larvae already irradiated for 12 h further decreased the C14 LC_50_ to 0.11 µM, corresponding to less than 1/4 of the value obtained after 1 h irradiation (Table 2).

**Table 2:** Median lethal concentrations (LC_50_) of C14 porphyrin.

hours of irradiation	LC_50_ (µM)	CI95%	R^2^	χ^2^
1	0.46	0.39–0.53	0.94084	0.29375
6	0.25	0.16–0.35	0.99381	0.03925
12	0.22	0.17–0.28	0.93035	0.39925
12 light+12 dark	0.11	0.08–0.13	0.94142	0.31300

C14 solutions at 7 increasing concentrations (range 0.03–4.3 µM) were incubated with 6 mg PFP at 28±2°C in the dark for 48 h. *Ae. aegypti* larvae (3^rd^–early 4^th^ instar, n = 100, 3 replicates) were introduced 12 hours before the beginning of the irradiation (1.0–4.0 mW/cm^2^).

#### Larvicidal efficacy of C14-loaded PFP

**Figure 7:**
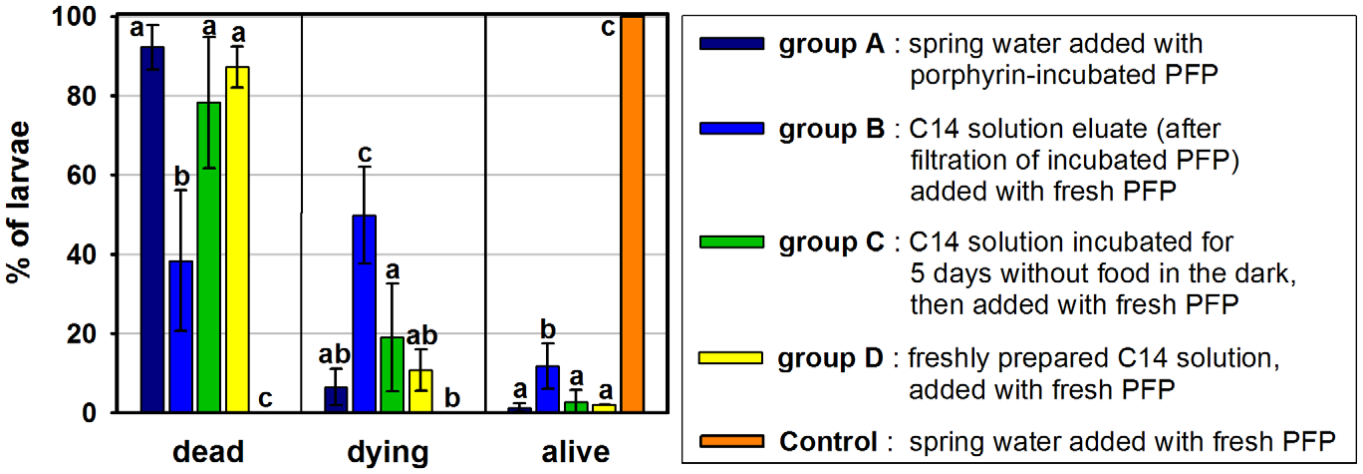
Larvicidal activities of C14 porphyrin-incubated and non incubated PFP. 5 µM C14 porphyrin solutions were used. PFP (70 mg), either pre-incubated with C14 or untreated, was added at the time of introduction of *Ae. aegypti* larvae (n = 50, see methods for details on preparation). Arithmetic means of percent dead, dying and living larvae after within 3 hours irradiation (intensity 1.0–4.0 mW/cm^2^). Error bars represent standard deviations, (n = 3 replicates of 50 larvae each). Lowercase letters above bars represent the results of LSD tests carried out independently for each category of larvae (see methods). Different letters above bars indicate statistically different means.

This experiment was carried out to investigate the route of intake and site of action of porphyrin C14 in the mosquito larvae. Larvae incubated in clean spring water added with PFP pre-incubated with the photosensitizing agent (group A) showed 92.2% mortality after irradiation (Fig. 7). No statistical difference was observed between this mortality level and the 87.1% and 78.2% mortalities achieved, respectively, by a freshly prepared C14 solution containing untreated PFP and a 5 µM C14 solution which had been incubated in the dark for 5 days before the introduction of untreated PFP (groups D and C). A lower mortality of 38.4% (*p*≤0.002) was observed in larvae exposed to the porphyrin solution “eluate”, i.e. the solution obtained by filtrating the PFP from its incubation medium (group B, see methods for details). In this treatment group, the highest percentage of dying larvae (49.8%; *p*≤0.002) was also observed, in contrast with all the other experimental groups where dying larvae amounted to 6.5%–19.1%. These mortality data confirm that C14 was loaded onto the “carrier” PFP, and show that C14 efficiently exerts its photosensitizing effect when adsorbed onto the PFP. The incubation solution, after being deprived of the C14-loaded PFP, has a lower C14 concentration and causes less mortality to the larvae. Incubating a C14 solution for five days in the absence of PFP resulted in a larvicidal medium that was equally effective as freshly prepared C14 solutions or C14-loaded PFP, indicating that the lower activity observed in the “eluate” (group B) is not due to the degradation of the porphyrin in water.

**Figure 8:**
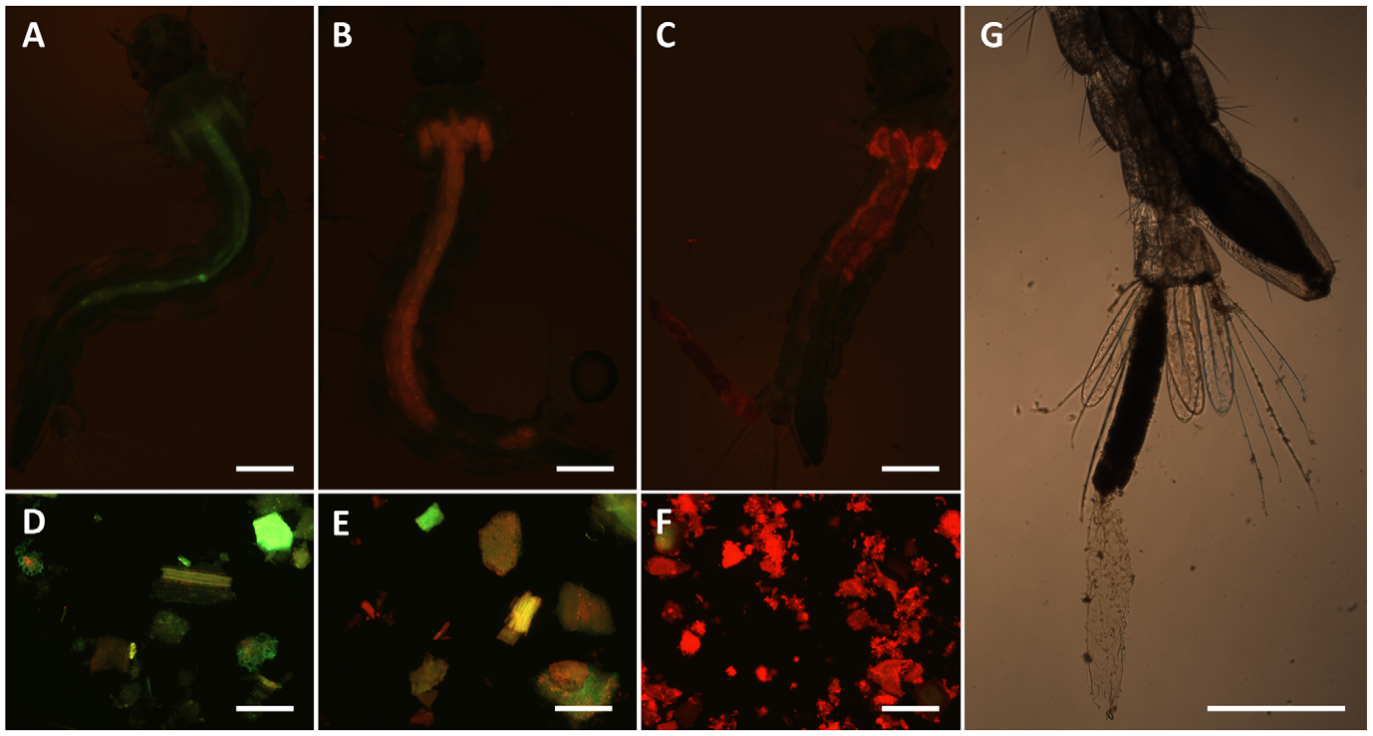
Detection of C14 porphyrin in exposed larvae and PFP particles. *Ae. aegypti* larvae were exposed to the photosensitizer at various conditions of food incubation. In particular, **A, D**: control; **B, E**: filtration eluate (experimental group B; see Fig. 6); **C, F**: typical example of larva and food particles from the other porphyrin treatments (experimental groups A, C and D; see Fig. 6). Larval food, either fresh or pre-incubated with porphyrin, was added in the trays at the same time of the introduction of the larvae, in all the groups. **G**: typical extrusion of the gut lining and content observed in C14 porphyrin-treated larvae (also visible in C). Scale bars represent 500 µm.

When photoexcited at 450–490 nm, C14 emits a red fluorescence which allowed for a qualitative assessment and comparison of the photosensitizer uptake by the larvae. In all the treated larvae, such fluorescence appeared to be limited to the midgut and the gastric caeca (Fig. 8). A strong fluorescence was observed in the midgut of larvae exposed to all the C14 treatments (experimental groups A, C and D; Fig. 8 C), exception made for the incubation eluate group, in which the larvae displayed a clearly less intense fluorescence in their gut and caeca (group B; Fig. 8 B). A mild green fluorescence was observed in control larvae, owing to the presence of untreated PFP particles in their midgut (Fig. 8 A). The presence/intensity of the C14 fluorescence pattern in the larvae matched what observed in PFP particles sampled from the corresponding larval incubation media (Fig. 8 D, E, F). These observations show that C14 converges into the digestive tract, and that the route of intake of the compound is by ingestion of C14-PFP complexes, even when the photosensitizing agent is initially dissolved in water. Treated and irradiated larvae were often found to expel fluorescent particulate material from the anus, which appeared to be enveloped by the gut epithelium or the peritrophic matrix, probably as a consequence of photoinduced damages to the digestive tract (Fig. 8 C, G).

#### Efficacy and residual activity of photolarvicidal formulations

The two experimental formulates C14PF-5 and C14PF-50 were found to contain 1.18 µg and 58.7 µg C14 porphyrin per mg of formulate, respectively. C14PF-50 maintained its larvicidal activity when incubated in trays containing spring water under the “natural” 12 h photoperiod of the climatic chamber (28±2°C; fluence rate 1.0–4.0 mW/cm^2^) for at least two weeks (Table 3). Conversely, C14PF-5 resulted devoid of any insecticidal activity, even just 48 hours after preparation. When C14 porphyrin was dissolved in water containing 6 mg of untreated PFP, a concentration-dependant residual activity was obtained: 1 week for 0.3 µM solutions and 2 weeks for 5 µM solutions. The absolute C14 amounts which the larvae had been exposed to are in agreement with the photolarvicidal activities observed (Table 3).

**Table 3:** Residual activity of C14 solutions and formulates on *Ae. aegypti* larvae.

	% mortality by incubation time*			
treatment	48 h	1 week	2 weeks	C14 (mg)/tray
control†	0 (0.0)	0 (0.0)	0 (0.0)	0
0.3 µM†	100 (0.0)	100 (0.0)	0 (0.0)	0.230
5 µM†	100 (0.0)	100 (0.0)	100 (0.0)	3.800
C14PF-5‡	0.3 (0.6)	0.3 (0.6)	0 (0.0)	0.007
C14PF-50‡	100 (0.0)	99.7 (0.6)§	99.7 (0.6)§	0.352

Mortality was assessed after 12 h irradiation (1.0–4.0 mW/cm^2^).

*time elapsed between the preparation of the trays and the introduction of 3^rd^–4^th^ instar larvae (n = 100 larvae; 3 replicates). During this period, trays were incubated in the climatic chamber (12 h photoperiod; 28±2°C; >90% RH). Numbers in parentheses indicate standard deviations.

**†:** trays contained 6 mg of untreated larval food in C14 porphyrin solutions at the indicated concentration.

**‡:** trays contained 6 mg of the indicated formulation in spring water.

**§:** one surviving larva was found in the tray. Such larvae were negative for C14 fluorescence at the microscope, therefore they hadn't fed during the experiment.

## Discussion

The *meso*-substituted C14 porphyrin appears to be photostable and to remain in the photoactive monomer form in aqueous solutions at concentrations up to 1 mM. Its singlet oxygen quantum yield of 0.46 is very close to the values previously found for a number of *meso*-substituted porphyrins [31], and denotes a high photosentisization efficiency. In fact, C14 elicited a strong phototoxic activity against 3^rd^–early 4^th^ instar larvae of the dengue vector *Ae. aegypti*, while lacking any intrinsic toxicity in the dark, as it is commonly the case for porphyrin photosensitizers [18]. Specifically, submicromolar concentrations of C14 caused a quick photo-mediated mortality of the larvae, at light intensities lower than 4.0 mW/cm^2^; the LC_50_ values were in the 0.1–0.5 µM range (i.e. 0.15–0.77 mg/l) for decreasing irradiation periods of 12–1 h. Porphyrin derivatives from natural sources have been previously shown to exhibit mosquito larvicidal action when dissolved in the water of larval containers under laboratory and semi-field conditions [24]–[27]. For instance, chlorophyllin and pheophorbid were shown to possess LD_50_ values of 6.88 and 8.44 mg/l, respectively, against *Culex* sp. larvae exposed for 3 h to a light intensity of 147 W/m^2^ [24], and hematoporphyrin determined 100% mortality in *Culex pipiens* larvae at a concentration of 10 µM after 3 days of exposure to natural sunlight [25].

The biological activity of porphyrin derivatives is known to be related to their molecular structure. Hydrophilic porphyrins are inefficient photoinsecticidal agents [34], possibly because of their inability to enter or cross the lipid layer of the cell membranes, which hinders their distribution into the target tissues. Hydrophobic porphyrins, on the other hand, can easily cross lipid membranes and reach different target cell compartments, such as plasma, mitochondrial and RER membranes [18], but may turn into photobiologically inactive dimers or oligomers in the aqueous medium, a major obstacle when targeting aquatic organisms such as mosquito larvae [26]. Amphiphilic porphyrins maintain a high tendency to cross the biological membranes and to partition in relatively large amounts within the tissues, owing to sufficient water solubility [35]. Therefore, such compounds show the most potent photokilling activities against insects such as flies and mosquitoes [24], [27], [34].

The amphiphilic character of C14 is guaranteed by the presence of four positive charges and a 14 carbon alkyl chain, hence it can undergo a ready and stable association to PFP particles , which are used to feed the mosquito larvae in the laboratory. We showed that C14 is taken up by ingestion of porphyrin-containing PFP. In agreement with our observations, Dondji et al. reported that incubating *Ae. aegypti* and *Anopheles stephensi* larvae in the dark in a hematoporphyrin solution (containing food) for 3 h and 24 h, respectively, enhanced the larval mortality at the following irradiation with visible light, in comparison to larvae exposed simultaneously to the photosensitizing agent and light [27]. Insect midgut has been identified as a major site of accumulation for several photosensitizers. In particular, *Ceratitis capitata* adult flies were shown to accumulate hematoporphyrin in the midgut, as well as in the fat body, malpighian tubules and cuticle after feeding on porphyrin-containing baits [36]. Treated and irradiated *Culex pipiens* larvae showed ultrastructural alterations at the level of the cuticle, fat body, muscle and midgut epithelium [37]. Damage to the peritrophic matrix was also described in the same study, an effect that could be linked with our observation of C14-treated larvae shedding their whole gut content. Larvae of the culicid *Eretmapodites quinquevittatus* accumulated porfimer sodium in the gastric caeca, gut, rectum, malpighian tubules and anal papillae [38]. *Aedes albopictus* larvae exposed to the non porphyric photosensitizers rose bengal, erythrosin B and α-terthiophene showed severe histological alterations of the midgut epithelium, fat body and malpighian tubules [39]. In our experimental conditions, however, fluorescence was not detected in other districts than the midgut and the gastric caeca, even in already dead larvae (data not shown).

Our fluorescence microscopy results indicate that C14 is released from PFP after its uptake, probably by digestion, then crosses the peritrophic matrix and diffuses in the ectoperitrophic space and in the caecal lumen. Both the peritrophic matrix and the caecal membrane are semi-permeable membranes which allow the passage of small molecules [40], and therefore they don't represent an obstacle for C14 diffusion. The singlet oxygen generated by C14 photoexcitation within the exoperitrophic space and caecal lumen can attack a large number of cell constituents, but possesses a short lifetime (in the microsecond range) and a mean pathway of less than 0.1 µm [41]. These features restrict the photooxidative damage to the immediate surroundings of the site where the singlet oxygen is generated and suggest that the phototoxicity exerted by C14 is limited to the midgut and caecal epithelia and may also involve the peritrophic matrix.

Porphyrins appear to be safe for humans at their photobiologically active doses. Porphyrin-based photodynamic therapy has been originally developed for the treatment of solid tumors [42] and is being successfully extended to several non-oncological pathologies, such as infectious diseases of microbial origin [43]. Moreover, porphyrins have been admitted as food additives [44], and toxicological studies have estimated that these dyes could induce important damage to humans only upon uptake of at least 100 mg/kg body weight [42], which is far greater than the amount required to generate an extensive toxicity in insects. The risks of phototoxicity on non target organisms, however, should not be overlooked. A 5 µM solution of C14 inhibits by 50% the formation of vegetative cells from cysts of the free-living ciliate *Colpoda inflata* even after 1 h incubation in the dark, and a similar inhibition level is obtained after just 5 min irradiation at a light intensity of 15 mW/cm^2^ [45]. A similar *meso-*substituted porphyrin, C12, has been recently tested on the two ecotoxicological crustacean models *Daphnia magna* and *Artemia franciscana*, showing opposite effects. While *D. magna* is highly photosensitized by the compound already at 0.3 µM, *A. franciscana* is remarkably resistant up to 10 µM in similar conditions [46].

The binding affinity of C14, and presumably of other amphiphilic porphyrins as well, to organic material can be exploited to develop cost-effective photolarvicidal bait formulates. By simple incubation in C14 solutions, we showed that PFP particles efficiently adsorb the photosensitizer. Six milligrams of our model formulation C14PF-50 and a 5 µM C14 solution directly used as a larval incubation medium were both 100% effective up to the longest time tested, i.e. two weeks, but the amount of C14 required to prepare the C14PF-50 was 10 times smaller than the C14 required to treat the incubation medium. This approach could provide several applicative advantages. *Ae. aegypti* often breeds in clean domestic water storages with little organic matter [47] and immature forms are often under nutritional stress in natural conditions [48]–[49]. A baited insecticidal formulate is therefore likely to be actively consumed by the larvae, hence allowing for an efficient and cost-effective employment of the photosensitizer, compared to direct dispersion of the compound in the breeding water. Furthermore, given their excellent safety profile, porphyrin-based photolarvicidal formulates appear to be compatible with application in household water storages for drinking and other domestic purposes. The presence of a photosensitizing insoluble formulate is not likely to affect the organoleptic properties of the stored water, and its ingestion can be easily avoided. These aspects may positively influence the acceptability of this larviciding approach by the recipient communities. Finally, the selection or manipulation of different porphyrin carriers allows to standardize the particle dimension to a size range that is especially palatable for mosquito larvae (e.g. 5–50 µm), thus reducing the risks of uptake by non-target organisms. This would permit the use of porphyrins in combination with natural predators otherwise susceptible to photosensitization. Additionally, by adjusting the physical properties of the formulate to conform to the typical larval feeding behavior (surface, column or bottom feeders) [50], it could be also possible to adapt this approach to the control of vectors of other diseases, e.g. *Anopheles* spp.

Semi-field and field studies are required to assess the potential of this approach in natural conditions. *Ae. aegypti* breeding sites are found both outdoors and indoors [4], [51]. While the photosensitization approach is expected to be effective on larvae located in outdoor containers, which are common productive breeding sites [49], [52]–[53], a major challenge is represented by breeding sites receiving little sunlight because of their shape or because of indoor or shaded location, a condition that for *Ae. aegypti* is not infrequent [54]–[55]. In a tropical setting, a container placed in a shady area may still receive enough indirect sunlight to trigger the photosensitization process, provided that the container itself is not too deep or covered with a lid. Preliminary measurements of light intensity were taken in Bobo-Dioulasso (Burkina Faso), 4–8 times a day for 27 days during July, September and October 2010, with weather conditions ranging between sunny and cloudy. The measurements revealed that the highest light intensity during the day reached 60–180 mW/cm^2^, depending on the weather conditions. In continuously shaded areas outdoors a light intensity of 1–9 mW/cm^2^ was recorded, which theoretically allows for an optimal photomediated toxicity, comparable to what described here in laboratory conditions. No quantitative data, to the best of our knowledge, is currently available on the average light intensity received by the various indoor and outdoor *Ae. aegypti* breeding sites in natural conditions, and this crucial aspect will have to be investigated in semi-field and field experiments.

The experimental formulate prototype described in this study was obtained using an inexpensive carrier material and simple methods. Porphyrin extraction and isolation from natural products, as well as their synthetic preparation (often by modification of natural porphyrins) [56] are relatively simple procedures. While an accurate estimation of the manufacturing costs of an optimized photoinsecticidal formulation is not possible at this stage, the cost of production of C14, currently 5 USD/Kg, is expected to keep the manufacturing cost of the final product at a low level. Several types of inexpensive natural and organic materials can be effectively used as carriers, such as ground animal food pellets, pollen granules or synthetic polymers (unpublished data from our laboratory).

Environmental management methods should represent the mainstay of vector control in dengue endemic areas and need to be encouraged and intensified [3]. In particular, interventions like improvement of water supply infrastructures, regular waste collection services and educational campaigns are crucial for the control of *Ae. aegypti* populations and hence dengue transmission [52], [57]. Chemical control through the use of larvicides, however, currently represents a valuable measure in many settings, and may benefit from the availability of new approaches and strategies, such as the bait-driven photosensitization described in this work, to complement its existing tools.

In conclusion, the meso-tri(N-methylpyridyl),meso-mono(N-tetradecylpyridyl)porphine (C14) is a potent photosensitizing agent, active at low doses and light intensities, which can be easily formulated into an effective and inexpensive bait-based photolarvicide. The laboratory studies reported in this work demonstrate the high potential of the photosensitization approach for the control of the larval stages of the dengue vector *Ae. aegypti*, and offer promising perspectives for the development of a new class of effective and safe disease vector control agents.
